# Entomological and Anthropological Factors Contributing to Persistent Malaria Transmission in Kenya, Ethiopia, and Cameroon

**DOI:** 10.1093/infdis/jiaa774

**Published:** 2021-04-27

**Authors:** Roland Bamou, Martin Rono, Teshome Degefa, Janet Midega, Charles Mbogo, Prophet Ingosi, Alice Kamau, Argaw Ambelu, Zewdie Birhanu, Kora Tushune, Edmond Kopya, Parfait Awono-Ambene, Timoléon Tchuinkam, Flobert Njiokou, Delenasaw Yewhalaw, Christophe Antonio Nkondjio, Joseph Mwangangi

**Affiliations:** 1 Organisation de Coordination pour la lutte Contre les Endémies en Afrique Centrale, Yaounde, Cameroon; 2 Vector-Borne Diseases Laboratory, Applied Biology and Ecology Research Unit, Department of Animal Biology, Faculty of Science, University of Dschang, Yaounde, Cameroon; 3 Kenya Medical Research Institute–Wellcome Trust Research Program, Kilifi,Kenya; 4 Center for Geographic Medicine Research Coast, Kenya Medical Research Institute, Kilifi, Kenya; 5 Pwani University Bioscience Research Centre, Kilifi, Kenya; 6 School of Medical Laboratory Sciences, Faculty of Health Sciences, Jimma University, Jimma, Ethiopia; 7 Tropical and Infectious Diseases Research Center, Jimma University, Jimma, Ethiopia; 8 Department of Environmental Health Sciences and Technology, Public Health Faculty, Jimma University, Jimma, Ethiopia; 9 Department of Health, Behavior and Society, Faculty of Public Health, Jimma University, Jimma, Ethiopia; 10 Department of Health Management, Institute of Health, Jimma University, Jimma, Ethiopia; 11 Laboratory of Parasitology and Ecology, Faculty of Sciences, University of Yaoundé, Yaoundé, Cameroon; 12 Vector Biology, Liverpool School of Tropical Medicine, Liverpool, United Kingdom; 13 Centre for Vector Disease Control, Kenya Medical Research Institute, Kwale,Kenya

**Keywords:** persistent malaria transmission, human behavior, malaria vectors, Kenya, Ethiopia, Cameroon

## Abstract

**Introduction:**

In order to improve our understanding of the fundamental limits of core interventions and guide efforts based on prioritization and identification of effective/novel interventions with great potentials to interrupt persistent malaria transmission in the context of high vector control coverage, the drivers of persistent disease transmission were investigated in three eco-epidemiological settings; forested areas in Cameroon, coastal area in Kenya and highland areas in Ethiopia.

**Methods:**

Mosquitoes were sampled in three eco-epidemiological settings using different entomological sampling techniques and analysed for *Plasmodium* infection status and blood meal origin in blood-fed specimens. Human behavioural surveys were conducted to assess the knowledge and attitude of the population on malaria and preventive measures, their night activities, and sleeping pattern. The parasitological analysis was conducted to determine the prevalence of *Plasmodium* infection in the population using rapid diagnostic tests.

**Results:**

Despite the diversity in the mosquito fauna, their biting behaviour was found to be closely associated to human behaviour in the three settings. People in Kenya and Ethiopia were found to be more exposed to mosquito bites during the early hours of the evening (18-21h) while it was in the early morning (4-6 am) in Cameroon. Malaria transmission was high in Cameroon compared to Kenya and Ethiopia with over 50% of the infected bites recorded outdoors. The non-users of LLINs were 2.5 to 3 times more likely to be exposed to the risk of acquiring malaria compared to LLINs users. Malaria prevalence was high (42%) in Cameroon, and more than half of the households visited had at least one individual infected with *Plasmodium* parasites.

**Conclusions:**

The study suggests high outdoor malaria transmission occurring in the three sites with however different determinants driving residual malaria transmission in these areas.

Despite the fact that >90% of malaria cases still occur in Africa, the burden of the disease distribution is largely heterogeneous across the continent [1]. Some countries such as Morocco are now free of malaria whereas others, including Cameroon, Nigeria, and Democratic Republic of the Congo, are classified as high-burden, high-impact countries because they have the highest contribution to malaria morbidity and mortality. Yet tremendous success has been achieved in disease control during the last decade. In many countries the disease prevalence has been reduced by half and several are heading toward the elimination phase. This success is considered to have resulted from the scale-up of key vector control interventions such as long-lasting insecticidal nets (LLINs) and indoor residual spraying (IRS) [2–4]. However, since 2014 the trend in disease reduction has stalled, with several regions reporting increased prevalence of malaria cases or no reduction at all in disease morbidity and mortality [4], suggesting that several factors may be limiting the efficacy of core interventions. Insecticide resistance is considered as a major threat undermining current core vector control interventions; several other factors are thought to be equally affecting the efficiency of these tools, but these factors have so far not been fully assessed [5–8].

Undertaking malaria vector surveillance could contribute important information regarding local vector population ecology, behavior, and dynamics after the scaling up of control interventions or could enable better appraisal of residual/persistent malaria transmission [8]. Several vector species have been reported to sustain residual/persistent malaria transmission including *Anopheles arabiensis* and *Anopheles gambiae* in Africa, *Anopheles dirus* in Southeast Asia, and *Anopheles albimanus* and *Anopheles darlingi* in the Americas [8]. Persistent malaria transmission is considered to be driven by outdoor biting and resting behavior of vectors, reduced or limited contact of mosquitoes with treated materials, or early exiting from houses or early-evening biting behavior [8–10]. Such vector behaviors could occur naturally or may result from insecticide-induced selective forces on malaria vectors (irritancy, repellence, and/or toxicity) [11]. Understanding factors contributing to the limited, or even attenuated performance of control interventions could enable in improving approaches for better disease prevention and control by these tools or provide knowledge for designing improved disease prevention and control tools. It is still not clear whether persistent malaria transmission is a consequence of mosquito feeding on humans in the absence of protection, including being indoors but not under nets, or being outdoors away from protected houses due to occupational, domestic, or recreational activities, or if vectors succeed to bite and transmit disease while people are still protected [5, 6].

In Africa, >20 species are considered as malaria vectors; however, a few are responsible for most of malaria transmission [12], and the predominant vectors vary from one eco-epidemiological setting to another. For instance in West Africa, vectors such as *An. gambiae*, *Anopheles coluzzii*, and *An. arabiensis* are the main vectors responsible for most malaria cases [13]. In Central Africa, species such as *An. gambiae*, *An. coluzzii*, *Anopheles funestus*, *Anopheles nili*, and *Anopheles moucheti* play a major role in malaria transmission [14–18]. In the coastal area of East Africa, the main malaria vector species include *An. gambiae*, *An. arabiensis*, and *An. funestus* [19–23], whereas in highland areas, species such as *An. arabiensis* and *Anopheles coustani* also play a major role in malaria transmission [23, 24]. Most species of the *An. gambiae* complex and *An. funestus* group have developed resistance to insecticides [15, 20, 25–28]. Other species such as *An. nili*, *An. moucheti*, and *An. coustani* are still susceptible to most insecticides [29], but they display high outdoor biting and resting behavior, which enable them to escape the current control interventions that target indoor feeding and resting behavior of mosquitoes [15, 16]. It is also not known whether persistent transmission of malaria results from the combination of the complexity of the vectorial system and poor coverage by LLINs and IRS or if this differs from one eco-epidemiological setting to another. In the present study, data collected from 3 sub-Saharan African countries with different eco-epidemiological features—forested areas of Cameroon, a coastal area of Kenya, and highland areas of Ethiopia—were assessed to better understand factors promoting persistent malaria transmission in different malaria-endemic foci in sub-Saharan Africa.

## METHODS

### Study Sites

**Figure 1.**
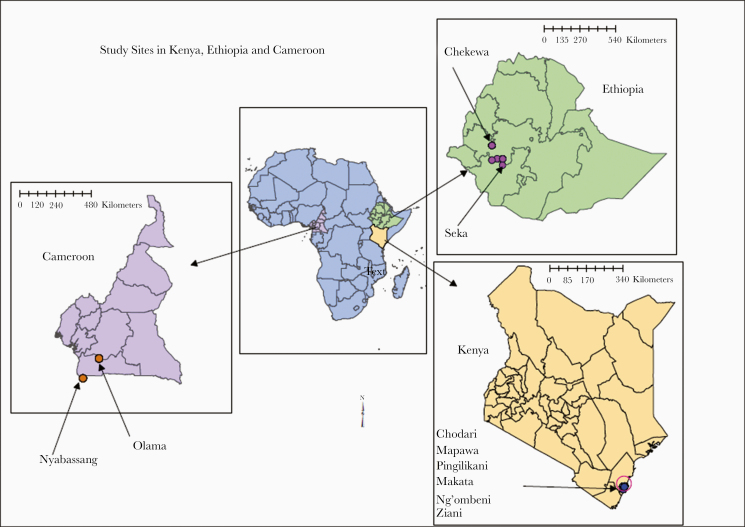
Map showing the study sites in the 3 sub-Saharan African malaria-endemic countries.

The study was conducted in 3 different eco-epidemiological settings of sub-Saharan Africa: Cameroon (forested area), Kenya (coastal area), and Ethiopia (highland area) (Figure 1 and Supplementary Table 1).

### Cameroon

The study was conducted in Olama (3°24′N; 11°18′E) and Nyabessan (2°80′N; 10°25′E) along the rivers Nyong and Ntem, respectively. They culminate at about 300–600 m above sea level. Both villages display high and perennial malaria transmission patterns and are located within the Congo-Guinean phytogeographic zone, characterized by a typical equatorial climate with 2 rainy seasons extending from March to June and September to November. Mean annual rainfall ranges from 1600 to 1800 mm [15] with daily temperature varying from 18°C to 28°C. Agriculture and fishing are the main socioeconomic activities of residents. Over the last decade, 3 (2004, 2011, and 2015), LLIN mass distribution campaigns have been conducted [30]. In 2019, the national malaria control program through the Ministry of Public Health launched the fourth LLIN distribution campaign. *Anopheles gambiae* sensu lato (s.l.), *An. nili*, and *An. moucheti* are the predominant malaria vectors in Nyabessan. In Olama, the main vector is *An. moucheti* followed by *An. gambiae* s.l. The coverage and usage of LLINs in the studied villages were high (approximately 96% and 90%, respectively).

### Kenya

The study was conducted in 6 villages: Chodari (3°7676′N; 39°7710′E), Ngombeni (3°7402′N; 39°7721′E), Mapawa (3°7402′N; 39°7721′E), Ziani (3°7484′N; 39°7207′E), Makata (3°7323′; 39°7965′E) and Pingilikani (3°7596′; 39°7895′E) along the coastal strip of Kilifi County in the coastal region of Kenya that is malaria endemic. Kilifi’s climate is considered tropical with precipitation mainly occurring during April through June and October through December. Mean annual precipitation ranges from 750 to 1200 mm [31]. *Anopheles gambiae* sensu stricto (s.s.), *An. arabiensis*, *Anopheles merus*, and *An. funestus* are the primary vectors of malaria and show a highly anthropophilic behavior [31]. The main socioeconomic activities are mainly fishing, farming, and tourism. The government of Kenya through the Ministry of Health has been distributing LLINs through a 3-year mass distribution cycle, and the last distribution was done in 2017. This was aimed at ensuring universal coverage in the region. LLIN coverage in 2016 in the study villages was 96.4%, and the usage rate was 78%.

### Ethiopia

The study was undertaken in Bore Tika (9°02′00′′N, 38°44′48′′E) and Chewaka (8°26′02′′E, 36°19′48′′N) villages in southwestern Ethiopia. Bore Tika is a village situated 350 km southwest of Addis Ababa in the suburb of Jimma town in Seka district at an altitude ranging from 1716 to 1752 m above sea level. The mean annual rainfall of the area is 1489 mm with temperature ranging from 11°C to 27°C. It is situated around Gilgel-Gibe river, which provides most of the mosquitoe breeding sites in the area. Chewaka village is situated in Chewaka district, Buno Bedele zone, Oromia Regional state, Ethiopia. Its altitude ranges from 1247 to 1262 m above sea level. The inhabitants are resettled communities from eastern Ethiopia due to recurrent drought. The population has a habit of spending early part of the night chewing khat (*Catha edulis*). The main socioeconomic activities of the community are mixed farming. Maize, sorghum, teff, khat, haricot beans, sesame, and pepper are widely cultivated. Coffee is an important cash crop in the area. In the country; in addition to IRS used as a major vector control tool since 1959, LLIN use has been scaled up since 2005, resulting in >64 million nets distributed (US President’s Malaria Initiative [PMI], 2016). In 2016, LLIN coverage and utilization in the study site were around 70.4% and 67.88%, respectively.

### House Selection

During the study, households where mosquito collection was conducted were randomly selected in each site and consent was obtained from the head of household. Houses were grouped according to trapping methods, that is, human landing catch (HLC; 10 houses), CDC light traps (10 houses), window exit traps (WETs; 5 houses) and backpack aspirators (10 houses). Other houses were used for pyrethrum spray catches (PSCs). Within the same sampling site, a distance of 50–100 m was maintained between houses and the number of homesteads sampled at each period of the study was dependent on the availability of traps.

### Mosquito Capture/Collection

Adult mosquitoes were collected from the selected houses using HLCs, CDC light traps, PSCs, WETs, and backpack aspirators. These sampling tools were used based on their availability in each setting.

### Human Landing Catches

Six to 10 houses were selected randomly in each of the selected villages per country for HLCs. In each of the 10 houses, indoor and outdoor mosquito collections were carried out from 6:00 pm to 6:00 am by 2 teams of 2 people per house using mouth aspirators. Outdoor mosquito collection was carried out about 8 m from each homestead/house selected for indoor mosquito collection by HLCs. The 2 teams (indoor/outdoor collectors) swapped positions in each homestead every hour of the night. Paper cups for mosquito collection were exchanged hourly following mosquito capture.

### Light Trap Catches

Ten houses were also selected for indoor and outdoor light trap catches from 2 study villages in each country in a similar way houses were selected for HLCs. Thus, the 10 houses served as sentinel stations for indoor and outdoor mosquito collection using LTCs. Mosquitoes were collected indoor and outdoor from 6:00 pm to 6:00 am from each selected house using standard CDC light traps (Centers for Disease Control and Prevention, Atlanta, Georgia). Traps were hung from the ceiling or from roof supports at the foot end of the bed where people sleep at night and the body of the trap was suspended about 1.5 m from the floor. Traps were also hung outdoor in the veranda or other outdoor structure for outdoor mosquito collection. Traps were set by trained research team members and run from 6:00 pm to 6:00 am. Collection bags were attached to the trap by stretching the open end of the bag over the bottom rim of the trap, and a label marked with the date and the site number was placed inside the collection bag. The number of people who slept in and animals kept inside the house the previous night were also recorded. Collection bags were retrieved from traps in each house in the morning from 7:00 am to 8:00 am by the collection team.

### Pyrethrum Spray Catches

Ten houses were randomly selected in each of the selected villages for spray sheet collection. Mosquitoes were collected from 6:00 am to 9:00 am. For this pyrethrum spray collection all the eaves, windows, and other exit points were closed or covered. White sheets were spread on the floor, aerosol insecticide was sprayed in the entire room, and the house was closed for 15 minutes after spraying. Prior to spraying, the heads of households were informed about the purpose and the time of spray and given clear instructions as to what they have to do before and after their houses have been sprayed. After 15 minutes, all of the knocked-down mosquitoes lying on the white sheets were collected carefully with forceps and placed in paper cups.

### Mosquito Collection Using Backpack Aspirator

Ten houses were also selected in each of the 2 selected sites in each country. Mosquitoes were collected from houses with human habitation, animal shed, and mixed habitation from 6:00 am to 9:00 am using backpack aspirator by trained collectors.

### Window Exit Traps

Window exit traps were performed in at least 5 different houses at least for 3 consecutive days per site per season. Traps were set at 6:00 pm and collected at 6:00 am the following day.

### Mosquito Identification

All mosquitoes collected were sorted into anophelines and culicines. All of the *Anopheles* mosquitoes were identified using taxonomic keys [31]. The *An. gambiae* complex, *An. funestus* group, and *An. moucheti* complex were further identified to sibling species using species-specific polymerase chain reaction (PCR). The wings and/or legs were used for sibling species identification [33, 34]. Genomic DNA was extracted from the legs and wings of subsamples of the collected specimens using the methods of Collins et al [35, 36] and amplified using specific diagnostic primers for *An. gambiae* s.s., *An. coluzzi*, *An. arabiensis*, *Anopheles amharicus*, and *An. merus* for *An. gambiae* complex [34, 37]. *Anopheles moucheti* was identified according to Kengne et al [38] using specific primers of *An. moucheti* subsp *moucheti*, *An. moucheti* subsp *nigeriensis*, and *An. moucheti* subsp *bervoetsi*. The protocol developed by Koekemoer et al [39] was used for molecular identification of member of the *An. funestus* group using specific primers of *An. leessoni*, *An. parensis*, *An. rivulorum*, and *An. vaneedeni.*

### Mosquito Processing

The cephalothoraces of all mosquitoes were tested separately for the presence of sporozoite of *Plasmodium falciparum* and/or *Plasmodium vivax* using enzyme-linked immunosorbent assay (ELISA) [40–42]. Fully blood-fed females collected by other collection methods other than HLC were also tested for blood meal sources by ELISA [43]. Blood meal sources were tested against human and bovine antibodies in all sites. In addition, goat and chicken antibodies were also used in Cameroon while only goat antibodies were added in Kenya.

### Human Behavioral/Occupational Factors Associated With Malaria Transmission

A semi-structured questionnaire was administered to participants in different sites to assess whether or not they sleep under their nets regularly, when they go to sleep and when they wake up in the morning, and leave their nets. In randomly selected houses, we targeted the parents (mothers and fathers) and an individual in the household who gave consent to participate in the study. The mothers gave information on their activities and those of their children in the morning and/or in the evening. Rapid diagnostic tests SD BIOLINE Malaria Ag. P.f./Pan (Standard Diagnostics, Republic of Korea) were used for malaria diagnosis for each respondent consenting to take part in the study to assess whether people reporting going to bed earlier and using nets are less affected by malaria than those going to bed late or not using nets. The association between the risk of malaria infection and the use of nets in each group was assessed using logistic regression analysis. The study was conducted twice a year during the dry and wet seasons in Cameroon and Ethiopia.

### Ethical Approval

This study was approved by the World Health Organization Ethics Review Committee (Protocol ID ERC.0002666), the Kenya Medical Research Institute Scientific Ethics Review Unit (KEMRI/SERU/CGMR-C/024/3148), the Cameroon National Ethics Committee for Research on Human Health (CNERSH) under the ethical clearance number 2016/01/685/CE/CNERSH/SP, and the Institutional Review Board of the College of Health Sciences, Jimma University (reference number HRPGG/269/2015). Written informed consent was obtained from the parents/guardians of the children during the malaria prevalence surveys, and assent was obtained for participants aged >13 years and <18 years.

### Data Analysis

Mosquito density was expressed as the mean number of mosquitoes of each species per trap per night. Calculation of the entomological inoculation rate (EIR) provided point measures of malaria transmission intensity. EIR is a commonly used metric that estimates the number of bites by infectious mosquitoes per person per unit time, which is a direct measure of malaria transmission intensity in an area. Sporozoite rates between mosquito species were compared using Fisher exact test.

### Biting Behavior/Cycle

To assess the behavior of malaria vectors, the indices proposed in Seyoum et al [44] were used. The mean proportion of bites on humans lacking a net that was obtained from a given vector population in the absence of any protective measure (*π*_*h,i*_) was calculated by weighing the mean indoor (B_i_) and outdoor (B_o_) biting rates for each hour of the night (t) by the proportion of humans reporting to have been indoors (I) and outdoors (1-I), respectively.


$$\pi_{h,i}=\underset{t=1}{\sum^{23}}({\text{B}}_{i,t}I_{t})/\underset{t=1}{\sum^{23}}\left({\text{B}}_{i,t}I_{t}+\text{ ​​}\;\text{​​}{\text{ B}}_{o,t}(1-I_{t})\right)$$

A univariate analysis was also carried out to independently determine whether any of the prospectively defined independent factors (age, sex, net usage, and sleeping behavior) was significantly associated with risk of malaria infection. To assess relative risk, odds ratios (ORs) with 95% confidence intervals (CIs) were computed using MedCalc Statistical Software version 15.8 (MedCalc Software, Ostend, Belgium; https://www.medcalc.org).

## RESULTS

### Mosquito Species Composition

**Figure 2.**
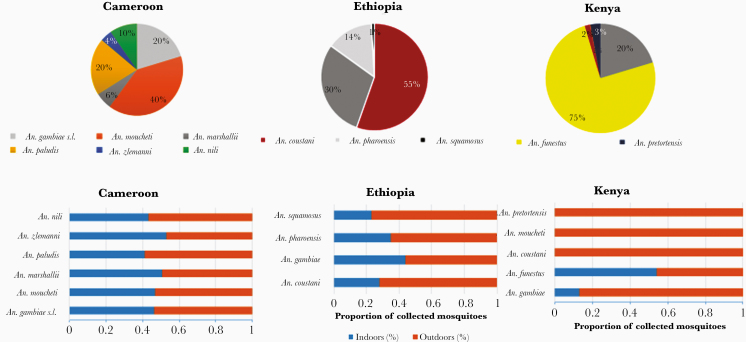
Anopheline mosquito species composition and behavior from Kenya, Ethiopia, and Cameroon.

The mosquito fauna collected in the 3 ecological/epidemiological settings (countries) was diverse and country specific (Figure 2; Supplementary Table 2). A total of 17 837 anophelines were collected. Anopheline mosquitoes collected in high densities in each site were considered as major vectors.

In the forested area of Cameroon, of the 15 925 anophelines collected, *An. moucheti* (n = 6344 [39.84%]) was the predominant malaria vector, followed by *An. gambiae* s.l. (n = 3232 [20.3%]). Other species included *Anopheles paludis* (n = 3202 [20.11%]), *Anopheles marshallii* (n = 942 [6%]), *An. nili* (n = 1538 [10%]), and *Anopheles ziemanni* (n = 667 [4%]). More than one-third of the collected *Anopheles* mosquitoes *(An. nili*, *An. paludis*, *An. ziemanni*, *An. coustani*, *An. marshallii*, *An. pharoensis*, *An. pretoriensis*, *and An. squamosus)* were secondary malaria vectors (n = 6349 [39.9%]). Molecular identification of *An. gambiae* complex using PCR revealed the presence of *An. gambiae* s.s. (n = 319 [72.17%]) and *An. coluzzi* (n = 123 [27.82%]) as members of the *An. gambiae* complex whereas molecular identification of *An. nili* (n = 40) and *An. moucheti* (n = 192) revealed the presence of *Anopheles ovengensis* (n = 40 [100%]) and *An. moucheti* subsp *moucheti* (n = 192 [100%]), respectively.

In the coastal area of Kenya, 415 *Anopheles* mosquitoes comprising 7 species were collected, of which *An. funestus* group (n = 311 [75 %]) was the predominant anopheline species, followed by *An. gambiae* s.l. (n = 84 [20%]). Other species included *An. pretoriensis* (n = 12 [2.9%]), *An. coustani* (n = 6 [1.4%]), *An. moucheti* (n = 1 [0.24%]), and *An. squamosus* (n = 1 [0.24%]). Molecular identification of *An. gambiae* complex showed that 80.95% (n = 68) belonged to *An. arabiensis* and 19.05% (n = 16) belonged to *An. gambiae* s.s.; *An. funestus* s.s. and *An. rivulorum* were found as members of the *An. funestus* group based on molecular identification.

Of the 2023 anopheline mosquitoes collected in the highlands of Ethiopia, *An. coustani* group (n = 945 [47%]) was the predominant anopheline species followed by *An. gambiae* s.l. (n = 852 [42%]). Those remaining (n = 226 [11%]) belonged to *An. pharoensis* and *An*. *squamosus*. Molecular identification of subsamples of *An. gambiae* complex revealed the presence of only *An. arabiensis*.

### Mosquito Behavior

Using HLC and CDC light traps, higher proportions of *Anopheles* mosquitoes were collected outdoors (9388/17 130; 54.80% [95% CI, 53.57%–55.92%]) than indoors (7742/17 130; 45.20% [95% CI, 44.2%–46.26%]) in all 3 countries. This outdoor host-seeking behavior is country dependent; it varies from 67% (95% CI, 62.63%–71.53%) in Ethiopia to 54% (95% CI, 53.19%–55.5%) in Kenya and Cameroon. It was also observed that higher density/proportion of *An. gambiae* s.l. was collected in all settings using PSC or aspirator (Prokopack). Other species such as *An. moucheti* were collected exiting houses in Cameroon (Figure 2; Supplementary Table 2). Of mosquitoes collected by Prokopack aspirator (Ethiopia), a high density/proportion of all anopheline mosquito species was collected from animal sheds compared with human and mixed habitation.

### Blood Meal Source

Freshly fed mosquitoes collected using sampling methods other than HLC were analyzed for their blood meal origin. It was observed that a high proportion of mosquitoes collected were blood-fed (>60%) except in Cameroon where only *An. gambiae* s.l. had a blood feeding rate exceeding 10%. In general, *An. gambiae* s.l., *An. coustani*, and *An. funestus* have high blood feeding rates. In Cameroon, the human blood index was very high (99%) with mixed (human and chicken) blood meal found in Ethiopia and Kenya. We detected blood meal from animal sources in 89.49% of the mosquitoes (bovine for Ethiopia and goat and bovine in Kenya) (Table 1). Mixed blood meals were observed in all settings, confirming the opportunistic behavior of most anopheline species such as *An. gambiae* s.l., *An. coustani*, *An. pharoensis*, and *An. funestus*. The blood meal source of about half of the Ethiopian mosquitoes was of unknown origin (unidentified) whereas only 14% and 3.3% were from unknown vertebrate sources in Cameroon and Kenya, respectively.

**Table 1.** Blood Meal Source of Malaria Vectors in Kenya, Ethiopia, and Cameroon

Country	*Anopheles* Species	Collected	Tested	BF Rate	Human	Goat	Bovine	H + C	H + B	H + G	G + B	H + B + G	Unknown
Ethiopia	*An. coustani*	168	116	69.05	0 (0)	NA	60 (51.7)	NA	4 (3.4)	NA	NA	NA	52 (44.8)
	*An. arabiensis*	486	327	67.28	4 (1.2)	NA	141 (43.1)	NA	23 (7.0)	NA	NA	NA	159 (48.6)
	*An. pharoensis*	96	44	45.83	0 (0)	NA	18 (40.9)	NA	1 (2.3)	NA	NA	NA	25 (56.8)
	Total	750	487	64.93	4 (0.8)	NA	219 (44.9)	NA	28 (5.7)	NA	NA	NA	236 (48.5)
Kenya	*An. funestus* s.s.	78	58	*74.36*	*8 (14.0)*	5 (9.0)	1 (2.0)	0 (0)	1 (2.0)	24 (41)	8 (14)	11 (19)	0 (0)
	*An. arabiensis*	15	12	*80*	0 (0)	2 (17)	0 (0)	0 (0)	1 (8)	4 (33)	2 (17)	2 (17)	1 (8)
	*An. pretoriensis*	2	0	*0*	0 (0)	0 (0)	0 (0)	0 (0)	0 (0)	1 (33)	1 (33)	0 (0)	1 (33)
	*An. gambiae* s.s.	0	0	*0*	0 (0)	1 (20)	0 (0)	0 (0)	0 (0)	3 (60)	0 (0)	1 (20)	0 (0)
	Total	95	62	*66.67*	8 (12.9)	8 (12.9)	1 (1.6)	0 (0)	1 (1.6)	32 (51.6)	11 (17.7)	14 (22.6)	2 (3.2)
Cameroon	*An. gambiae* s.l.	355	100	28.17	77 (77)	0 (0)	0 (0)	3 (3)	0 (0)	0 (0)	0 (0)	0 (0)	20 (20)
	*An. nili*	59	5	8.47	5 (100)	0 (0)	0 (0)	0 (0)	0 (0)	0 (0)	0 (0)	0 (0)	0 (0)
	*An. moucheti*	313	25	7.99	25 (100)	0 (0)	0 (0)	0 (0)	0 (0)	0 (0)	0 (0)	0 (0)	0 (0)
	*An. paludis*	175	4	2.29	4 (100)	0 (0)	0 (0)	0 (0)	0 (0)	0 (0)	0 (0)	0 (0)	0 (0)
	Total	902	134	14.86	111 (82.8)	0 (0)	0 (0)	3 (2.2)	0 (0)	0 (0)	0 (0)	0 (0)	20 (14.9)

Data are presented as No. (%) unless otherwise indicated. Mosquito species shown in italics.

Abbreviations: BF, blood feeding; G + B, goat plus bovine blood; H + B, human plus bovine blood; H + B + G, human plus bovine plus goat blood; H + C, human plus chicken blood; H + G, human plus goat blood; NA, not available; s.l., sensu lato; s.s., sensu stricto.

## MOSQUITO BITING ACTIVITY AND SLEEPING BEHAVIOR (INDOOR AND OUTDOOR EXPOSURE TO MALARIA INFECTION)

**Figure 3.**
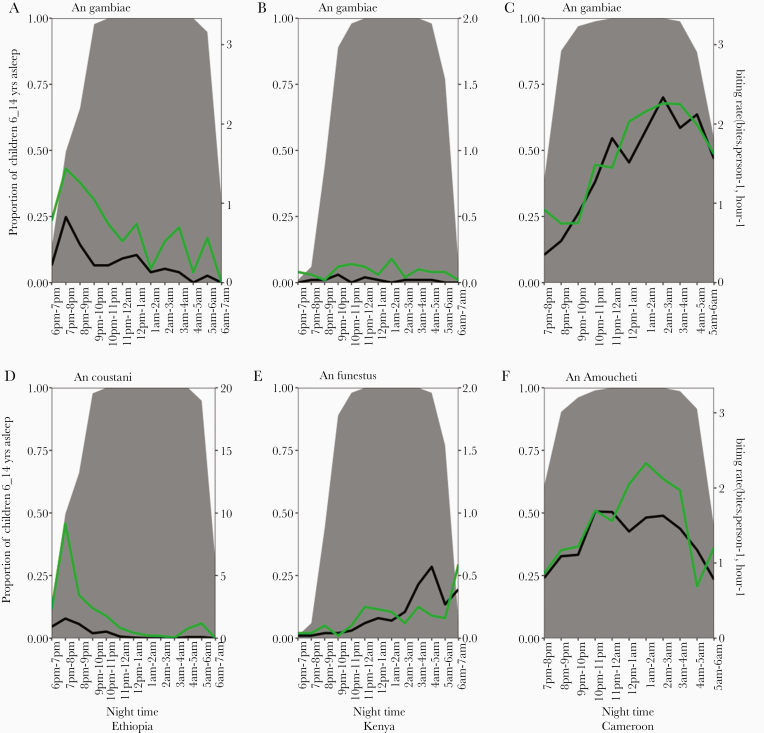
Hourly biting activity of *Anopheles* mosquitoes both indoors (black lines) and outdoors (green lines) for the *Anopheles* vector and *Anopheles gambiae* sensu lato. Gray shading represents the proportion of children aged 6–14 years asleep at each hour of the night.

More than 70% of children aged 6–14 years were reported to be asleep between 6:00 pm and 9:00 pm; this rose monotonically over the course of the night, reaching 100% by 10:00 pm in all 3 countries (Figure 3). Similar trends were observed in children aged <5 and >15 years (Supplementary Data). However, up to 50% of adult household members (fathers and mothers) were awake after 9:00 pm with the latest sleeping at around midnight. The timing of human activity, both indoors and outdoors, may modulate vector-human contact. For example, in Ethiopia, peak biting activity for both *An. coustani* group and *An. gambiae* s.l. occurs early in the evening (6:00–8:00 pm) before children go to sleep under the protection of insecticide-treated nets (ITNs), while in Cameroon, both *An. moucheti* and *An gambiae* s.l. peak biting occurs between 10:00 pm and 2:00 am, when a majority of household members are under the protection of ITNs (Figure 3). In Kenya, a stable biting activity is observed for indoor feeding vectors; however, for outdoor feeding and resting mosquitoes, especially *An. funestus* group, biting activity gradually increases throughout the night with peak activity between 3:00 am and 5:00 am.

### Indoor and Outdoor Exposure of Malaria Infection

Using Seyoum’s formula, the proportion of exposure occurring indoors/outdoors was estimated [45]. In general, indoor exposure (*πi*) was 0.82, 0.27, and 0.84, respectively, in Cameroon, Ethiopia, and Kenya. Indoor exposure of mosquito varies with human population age, mosquito species, and country. In Cameroon, *πi* for *An. gambiae* was 0.84 while exposure to *An. moucheti* was 0.81. In Ethiopia, most species bite outdoor with high proportion of people outdoors during the night; *πi* was 0.48 and 0.2 for *An. gambiae* s.l. and *An. coustani*, respectively. In Kenya, *πi* was 0.47 and 0.9 for *An. gambiae* s.l. and *An. funestus*, respectively.

Between 7:00 pm and 9:00 pm (early evening), 18.31%, 85.67%, and 91.90% of exposures occurred outdoors in Cameroon, Ethiopia, and Kenya, respectively. However, from 10:00 pm to 4:00 am, nearly all exposures occurred indoors, while between 4:00 am and 6:00 am, outdoor/indoor exposure was high in all settings except in Kenya (45.76% in Cameroon, 40.66% in Ethiopia, and 4.87% in Kenya).

### Indoor and Outdoor Malaria Transmission Intensity

A total of 14 140 *Anopheles* mosquitoes (12 802 from Cameroon, 1022 from Ethiopia, and 316 from Kenya) collected by HLC were tested for *Plasmodium* circumsporozoite protein (CSP) using ELISA. In Cameroon, the indoor and outdoor sporozoite rate was 1.4 and 1.6%, respectively, with an overall sporozoite rate of 1.5%. Infection rates for *An. gambiae* s.l., *An. moucheti*, *An. nili*, *An. paludis*, *An. zeimanni*, and *An. marshali* were 6.0%, 0.7%, 0.9%, 0.7%, 0.3%, and 0.2%, respectively. In Ethiopian sites, the sporozoite rate for *An. gambiae* s.l. and *An. coustani* was 0.5% and 0.2%, respectively, all being from outdoor collections. The fact that no infection could be detected indoors could be linked to the limit of detection with the sample size used. None of the tested *An. pharoensis* and *An. squamosus* specimens from Ethiopia were positive for CSPs. In Kenya, the indoor and outdoor sporozoite rate was 3.7% and 2.7%, respectively, with an overall sporozoite rate of 3.0%. *Anopheles gambiae* s.l., *An. funestus* group, and *An. pretoriensis* contributed to infection rates in Kenyan sites (Table 2).

**Table 2.** Malaria Transmission Intensity in Cameroon, Ethiopia, and Kenya

*Anopheles* Species	Biting Locations	HBR (Bites/Person/Night)			Sporozoite Index, %^a^			EIR (Infective Bites/Person/Year)		
		Cameroon	Ethiopia	Kenya	Cameroon	Ethiopia	Kenya	Cameroon	Ethiopia	Kenya
*An. gambiae* s.l.	Indoor	5.22	3.13	0.17	0.05 [0.04–0.06] (1171)	0.000 […]	0 (11)	95.28	0.00	0.00
	Outdoor	6.35	8.61	0.85	0.06 [0.05–0.08] (1004)	0.005 [0.004–0.01]	0.018 [0.0–0.1] (55)	139.04	15.87	5.63
	Total	5.78	5.87	0.508	**0.06** [0.05–0.07] **(2175)**	**0.004** [0.002–0.005]	**0.015** [0.0–0.08] **(66)**	**126.69**	7.93	**2.815**
*An. moucheti*	Indoor	10.72	0.00	0.00	0.008 [0.004–0.011] (2604)	NA	NA	31.32	NA	0.00
	Outdoor	12.65	0.00	0.02	0.006 [0.0033–0.009] (2545)	NA	0 (1) […]	27.71	NA	0.00
	Total	11.69	0.00	0.008	**0.007** [0.005–0.009] **(5149)**	NA	**0 (1)** […]	**29.86**	NA	**0.00**
*An. funestus* group	Indoor	0.00	0.00	1.86	NA	NA	0.04 [0.01–0.09] (121)	0.00	NA	28.15
	Outdoor	0.00	0.00	1.71	NA	NA	0.03 [0.01–0.09] (111)	0.00	NA	16.89
	Total	0.00	0.00	1.785	NA	NA	**0.03** [0.02–0.07] (232)	**0.00**	NA	22.52
Other species	Indoor	10.64	6.13	0.00	0.005 [0.003–0.008] (2765)	0.000 […]	0 (0)	19.42	0.0	0.00
	Outdoor	13.47	26.57	0.26	0.007 [0.004–0.011] (2713)	0.002 [0.0–0.01]	0.058 [0.001–0.33] (17)	34.42	15.9	5.63
	Total	12.06	16.35	0.13	**0.006** [0.004–0.008] **(5478)**	**0.001** [0.0–0.01]	**0.058** [0.001–0.33] **(17)**	**26.40**	7.9	**2.81**
All species	Indoor	26.59	9.26	2.03	0.014 [0.011–0.017] (6540)	0.000 […]	0.037 [0.01–0.08] (132)	135.85	0.00	28.15
	Outdoor	32.47	35.17	2.83	0.016 [0.013–0.019] (6262)	0.002 [0.001–0.01]	0.027 [0.01–0.06] (184)	189.64	31.74	28.15
	Total	29.53	22.22	2.43	**0.015** [0.013–0.017] **(12 802)**	**0.002** [0.001–0.01]	**0.03** [0.015–0.05] **(316)**	**161.67**	15.87	**28.15**

Abbreviations: EIR, entomological inoculation rate; HBR, human biting rate; NA, not available; s.l., sensu lato.

^a^Sporozoite Index shown as percent of plasmodium positive mosquitoes and number tested and compared by Fischers exact test.

The overall annual indoor entomological rates (EIRs) for Cameroon, Ethiopia, and Kenya were 135.85, 0, and 28.15 infective bites per person per year, respectively, whereas the annual outdoor EIRs for the 3 sites were 189.64, 31.74, and 28.15 infective bites per person per year, respectively.

## CONTRIBUTION OF OUTDOOR BITING BEHAVIOR AND SECONDARY/MINOR MALARIA VECTORS IN MALARIA TRANSMISSION

In these 3 countries, *An. moucheti*, *An. gambiae* s.l., and *An. funestus* group are defined as major vectors while others (*An. paludis*, *An. ovengensis*, *An. marshallii*, *An. ziemanni*, *An. pretoriensis*, *An. coustani*, *An. pharoensis*, and *An. squamosus*) are defined as secondary or minor vectors. The contribution of these vectors to malaria transmission and the outdoor biting behavior was estimated from the total transmission index (EIR). In Cameroon, 58.24% of the total transmission occurs outdoors while secondary vectors contribute to 13.68% of total transmission. In Kenya, 50% of transmission occurs outdoors while secondary vectors contribute to <10% of the transmission (7.1%). Surprisingly, all transmission occurs outdoors in Ethiopia while secondary vectors contribute to 50% of the total transmission occurring in the country.

## SLEEPING BEHAVIOR AND ACTIVITIES KEEPING PEOPLE OUTDOORS IN THE EVENING

**Figure 4.**
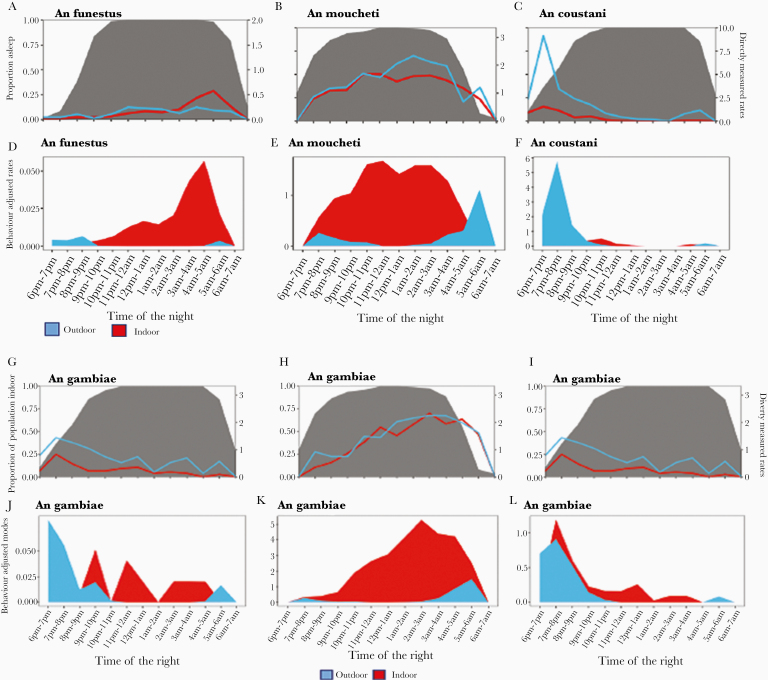
Exposure risk to indoor and outdoor mosquito bites with age category in Ethiopia, Cameroon, and Kenya. A1, A2, and A3 represent directely measured human biting rate and human location for *Anopheles gambiae* sensu lato in Ethiopia, Cameroon, and Kenya, respectively. B1, B2, and B3 represent behavior-adjusted biting rate for an individual for *An. gambiae* and C1, C2, and C3 for *Anopheles coustani*, *Anopheles moucheti*, and *Anopheles funestus*, respectively, for Ethiopia, Cameroon, and Kenya.

Sleeping patterns varied between ecological settings (Figure 4). The proportion of people staying outdoors was high in the evening (6:00–8:00 pm) with the majority going to bed after this period. Except in Ethiopia where the household head (father) went asleep early while children and mother went to bed late (by 9:00 pm), most fathers and school-aged children were late-night sleepers. In most cases, early morning/outdoor activities began at 4:00 am. Different activities were observed and justified to maintain these outdoor activities and the activities were sex, age, and country specific. In most cases, mothers were engaged in preparing food/coffee or taking care of babies with the help of female adolescents, while fathers and male children took care of the animals, read Quran, and studied their lessons. Also, it was observed in Cameroon and Kenya that fathers chat in the evening while drinking alcohol (palm wine or beers). In Cameroon, watching TV (football matches) or playing “songo” or selling of food and alcoholic beverages in the evening were the most common activities keeping adolescents outdoor.

It was observed in Cameroon that time spent outdoors increased during popular games such as football (European champions league) or social ceremonies such as wedding, burials, funerals, parties, festivities, or toward the end of each month when people get their salary.

## CHARACTERISTICS OF THE STUDY POPULATION

Sociodemographic characteristics of the study population were country dependent. House walls were constructed mainly from wood and mud in Cameroon, and with mixed materials in Ethiopia and Kenya. Iron sheeting was the main roofing material in Ethiopia and Cameroon whereas grass was the main material in Kenya. It was common to see between 1 and 5 persons per house in all the sites. Bed net ownership (percentage of houses having at least 1 net) was high in all sites with 96.8%, 70.4%, and 96.7%, respectively, in Cameroon, Ethiopia, and Kenya; utilization (proportion who slept in the net the previous night) was also high—67.88%, 94.54%, and 78% in Ethiopia, Cameroon, and Kenya, respectively (Table 3).

**Table 3.** Sociodemographic and Socioeconomic Characteristics of Surveyed Populations in Kenya, Ethiopia, and Cameroon

Characteristic	Kenya		Ethiopia		Cameroon	
	No.	(%)	No.	(%)	No.	(%)
No. of households	304	…	274	…	186	…
LLIN ownership	294	(96.7)	193	(70.4)	180	(96.8)
LLIN usage	294	(78)	186	(67.88)	178	(95.7)
Sex of head of household						
Male	114	(37. 5)	86	(31.4)	105	(56.45)
Female	190	(62.5)	188	(68.6)	81	(43.55)
Age of head of household, y						
15–34	103	(36.14)	150	(58.37)	78	(47.56)
35–54	111	(38.95)	82	(31.91)	64	(39.02)
≥55	71	(24.91)	25	(9.73)	28	(17.07)
No. of household members						
1–5	184	(61.33)	172	(63.24)	78	(41.9)
6–10	113	(37.67)	89	(32.72)	74	(39.8)
≥11	3	(1.00)	11	(4.04)	34	(18.3)
No. of household members aged <5 y						
1–5	242	(100)	97	(100)	110	(59.14)
6–10	0	(0)	0	(0)	7	(3.8)
≥10	0	(0)	0	(0)	2	(1.07)
No. of rooms per household						
1–5	…	…	252	(97.30)	128	(68.82)
6–10	…	…	7	(2.70)	58	(31.18)
≥10	…	…	0	(0)	3	(1.61)
Main wall material						
Brick	8	(2.66)	0	(0)	6	(3.2)
Cement/paint	0	(0.00)	0	(0)	15	(8.1)
Mud plastered	0	(0.00)	0	(0)	28	(15.1)
Wood	16	(5.32)	…	(0)	73	(39.2)
Mixed	267	(88.70)	261	(100)	…	…
Mud	0	(0.00)	0	(0)	64	(34.4)
Stone	10	(3.32)	0	(0)	0	(0)
Main roof material						
Cement	0	(0.00)	0	(0)	1	(0.55)
Grass	220	(77.46)	8	(2.99)	4	(2.18)
Iron sheet	64	(22.54)	260	(97.01)	178	(97.27)

No. is the number or size of each group; % is the proportion (number in each group divided by the total).

Abbreviation: LLIN, long-lasting insecticidal net.

## DETERMINANTS OF MALARIA PREVALENCE

In this part, only Cameroon collected data accurately (see Table 4).

**Table 4.** Risk of Malaria Infection by House Type, Sleeping Behavior, and Population Age in Cameroon

Characteristic	Prevalence^a^	OR (95% CI)	*P* Value
Sleeping behavior			
Sleep late	(30) 69/229^b^	0.57 (.41–.80)^c^	**<.001**
Sleep early	(43) 255/569	1	
Wake up early	(38) 57/150	0.89 (.62–1.29)	.72
Wake up late	40) 267/675	1	
House type			
Cement	(33) 61/185	1	
Wood	(53) 188/355	2.29 (1.58–3.32)	**<.0001**
Mud	(26.9) 73/271	0.75 (.5–1.13)	.16
Age, y			
[0–5]	(42) 126/300	1	NA
[5–10]	(53) 128/240	1.58 (1.12–2.22)	**.008**
[11–15]	(42) 63/151	0.98 (.66–1.47)	.95
>16	(12) 18/145	0.2 (.11–.34)	**.000**
Use of LLIN			
Yes	(35.31) 220/623	1	
No	(54.32) 88/162	2.17 (1.53–3.09)	**<.0001**

The values in bold show significance (*P* Value).

Abbreviations: CI, confidence interval; LLIN, long-lasting insecticidal net; OR, odds ratio.

^a^Malaria Prevalence.

^b^number infected/number screened.

^c^95% CI.

### Age-Specific Malaria Prevalence

A total of 836 individuals (430 in Olama and 406 in Nyabessan) were tested with a malaria rapid diagnostic test (RDT). Of this population, 351 were found to be infected with *Plasmodium* species, representing a prevalence of 42%. The prevalence of infections was high in the age group 5–10 years. Children aged <16 years have a high risk of malaria infection.

### House Type and Malaria Prevalence

A total of 258 houses were visited for malaria detection during the study. The proportion of people detected with malaria parasites was high, at 57.36% (95% CI, 51.67%–78.25%). The prevalence of malaria infection in the inhabitants varied significantly according to house characteristics (χ ^2^ = 4.58; *P* = .032). People living in cement houses were found to have fewer malaria parasite infections compared to others living in mud or wood houses.

### LLIN Utilization and Malaria Prevalence

RDT was used to detect malaria infection in people. It appears that people not using LLINs were twice more at risk of malaria infection (OR, 2.17 [95% CI, 1.53–3.09]) as compared to users.

## DISCUSSION

The study objectives were to document entomological and anthropogenic behavioral factors, which could contribute to persistent malaria transmission in 3 settings with high coverage with LLINs or IRS and with different ecological features across sub-Saharan Africa: Central African equatorial forest region (Cameroon), East African coastal area (Kenya), and East African highlands (Ethiopia).

Persistent transmission of malaria was recorded in the 3 sites and was in conformity with continuous malaria transmission occurring in these areas. Yet the EIR rate was different from one country to the other and this could be due to different malaria burden in the 3 sites [2]. These differences in malaria transmission may be due to the difference observed in the level of outdoor mosquito biting across the 3 countries, which may likely result in differences in persistent/residual transmission and the effectiveness of current malaria prevention actions. Surprisingly, the high outdoor biting behavior of malaria vectors from Ethiopia was not associated with high malaria transmission and may be due to the low proportion of the major vector *An. gambiae* s.l. Study sites in Cameroon display the highest EIR followed by Kenya and Ethiopia. The following could suggest different protection provided by indoor-based control tools (treated nets and/or IRS) or different efficiency/competence of major vectors in the area. In Cameroon, vector populations were found to be highly anthropophilic compared to Kenya and Ethiopia. This close reliance on human blood could be a major factor increasing the risk of malaria transmission. Yet in all the sites, malaria transmission was high outdoors compared to indoors. Despite this high transmission, estimates weighted with human behavior as described in Monroe et al [46] showed high exposure risk indoors in all sites except in Ethiopia, where high proportions of mosquitoes and population are found outdoors during the night. The proportion of bites preventable by control tools such as LLINs was not estimated during this study and could constitute a limitation [46, 47].

Up to 15 species were recorded during the study period, consistent with the high diversity of the anopheline fauna across Africa [12]. *Anopheles gambiae* s.l. was recoorded as the major malaria vector in all settings, contributing for >90% of the total transmission. Apart from *An. gambiae* s.l., which was present in the 3 sites, differences in species composition and abundance were recorded between the 3 settings and were in accordance with the peculiarity of each site [12]. In Cameroon the main vectors were *An. gambiae* s.l. and *An. moucheti*. These species were responsible for >90% of malaria transmission cases in the forest region; these findings are consistent with previous studies in the area [15, 48]. Other species such as *An. paludis* and *An. ovengensis* were also recorded infected and involved in malaria transmission as reported in earlier studies [15–17, 48]. In Kenya, vectors collected included *An. gambiae* s.l. and *An. arabiensis*. These are well-known vectors in the country [23, 49–53]. In Ethiopia, 2 anopheline species, *An. gambiae* s.l. and *An. coustani*, were found to be infected with *Plasmodium* species. These species are known to display different biting, feeding, and resting behaviors [54–56]. Although Ethiopia is situated in a highland area, the malaria situation in the country is largely complex with both *P. falciparum* and *P. vivax* as the main parasites together with the presence of new vectors such as *Anopheles stephensi* in the country.

Studies conducted in the 3 sites indicated high exophagic behavior of mosquitoes. The following could suggest high influence of treated nets that could, through their excito-repellency effect, reduce the entry of mosquito indoors or could be due to a change of behavior of mosquitoes in relation with human activities or genetic changes [57].

Improving malaria control strategies also requires a good understanding of when and where people are most likely to be exposed to mosquito infective bites. In addition to entomological surveys, human population surveys were conducted to link human activities to the mosquito biting cycle. A majority of people return home by 10:00 pm and wake up by 4:00–5:00 am; this behavior makes exposure to mosquito infective bites preventable if we assume that they use LLINs at night and that anopheline bites mostly occur in the middle of the night when people are asleep. However, as reported elsewhere [5, 6, 58, 59], different social or livelihood activities that lasted throughout the night, such as feasts, burials, and commemorations, could disrupt usual sleeping patterns and reduce the use of LLINs during the night. In Kenya and Ethiopia, it appeared that high anopheline biting densities outdoors were recorded between 6:00 pm and 9:00 pm, whereas in Cameroon a high outdoor biting rate was recorded instead between 4:00 am and 6:00 am. The latter could result from the fact that many people wake up in the early hours to prepare for work or to do some domestic activities before going to work or school. Similar behavior has been reported elsewhere [6]. Although a certain number of similar activities was found to maintain people outdoors in the 3 countries (eg, cooking, fetching water, eating, and relaxing/chatting), a certain number of peculiarities was also observed. In Ethiopia, for example, herding cattle or sharing sleeping quaters with cattle was also recorded, and this could have increased the risk of being bitten by zoophilic mosquito species. Mosquitoes such as *An. coustani* displaying a high opportunistic behavior were recorded to be frequently infected in the area. In Kenya, where people sleep in the same house with cattle, different species were found to be infected with malaria parasites. In Cameroon, despite the high anthropophilic behavior of mosquitoes, a change in the biting activities of vectors closely associated with human behavior was recorded. *Anopheles gambiae* and *An. moucheti*, the main vectors in the region, were found to display high biting rates early in the evening or early in the morning when people were out of the house conducting a certain number of activities.

The main vector tools used by the population in the different study sites included IRS and LLINs; these tools are efficient when people are indoors or asleep in bed. People working outdoors are considered to be vulnerable to mosquito infectious bites. No additional tools to target outdoor biting mosquitoes were used by the population in either sites. These observations have similarly been reported in Tanzania where the use of repellent was not observed during outdoors activities [6, 60–63]. The absence of tools that could protect people from mosquito bites outdoors is a critical gap in vector control measures that needs to be covered to reduce the risk of malaria transmission, particularly in the context of malaria elimination.

High malaria prevalence in the human population was observed in Cameroon despite high bed net usage. The following could be due to the influence of different factors such as sleeping time, the number of people residing within a house, and the proportion of people using bed nets or the type of house design/construction. Unexpectedly, people going to bed early were twice likely to be infected than other age group. The waking up time in the morning was not associated with a risk of malaria transmission; this could be due to the limited power of analysis as not everyone responded to this question. Children aged 5–10 years were more likely to be infected than the older age groups. Nonusers of ITNs were also found to be twice more infected by *P. falciparum* than ITN users [15], highlighting the importance of ITN use despite the increased prevalence of insecticide resistance in vector populations, which must be preserved to improve the control of malaria. The misuse of control tools by residents may hinder the fight against malaria. There is a need for continuous sensitization of the population to improve bed net usage and malaria prevention. House characteristics also appeared to be an important risk factor, but this has so far not been deeply assessed. It is possible that stopping collection at 6:00 am might have underestimated the exposure rate to mosquito bites because a substantial proportion of exposure could be taking place after 6:00 am, as highlighted elsewhere [46, 47]. Poor house construction was associated with increased malaria transmission risk. Previous studies also established a direct relationship between house characteristics and malaria transmission [64–66].

## CONCLUSIONS

Although there is proper use of LLINs in the equatorial forest, highland, and coastal regions in Africa, effective in preventing indoor malaria transmission, people still complain about the persistence of disease transmission. Malaria risk/transmission was found to be higher outdoors, during early evening (before bed time) or early morning, and during social activities. High transmission was found in the equatorial forest where a high density of *An. gambiae* s.l., a primary vector in Africa, was found. Secondary vectors also contribute to malaria transmission. These observations suggest that complementary tools to LLINs are still needed to protect people when outdoors if malaria elimination is still on the agenda. These findings highlight the need to search additional means to control malaria transmission mainly vectored by outdoor-seeking mosquitos and other related diseases in these foci where the population could be exposed to the risk of outbreaks due to the transmission of pathogens from primates to humans. As human behavioral activities exposing populations to malaria risk are similar to those found in other countries in Africa (Tanzania) and elsewhere (Vietnam), addressing these issues with existing tools could be difficult. New personal protection tools that do not rely on mosquito biting behavior will be needed in the arsenal of control tools. In addition, the use of an integrated vector control approach to improve the performance of LLINs and limit the expansion of insecticide resistance could be indicated. In addition, more sensitization needs to be done within endemic regions to promote the use of disease control measures and to avoid human activities that increase the risk of exposure and transmission of malaria.

## Supplementary Data

Supplementary materials are available at *The Journal of Infectious Diseases* online. Consisting of data provided by the authors to benefit the reader, the posted materials are not copyedited and are the sole responsibility of the authors, so questions or comments should be addressed to the corresponding author.

jiaa774_suppl_Supplementary-Table-S1

jiaa774_suppl_Supplementary-Data_Caption

## Notes

***Author contributions.*** D. Y., C. A. N., and J. M. conceived and designed the study, contributed to tool and protocol development, helped manage data collection, advised on data analyses and interpretation, and edited the manuscript. B. R. managed entomological field data collection, performed epidemiological studies and laboratory analysis, analyzed results and data interpretation, wrote the first draft of the manuscript, and wrote the final manuscript. M. R. and T. D. managed entomological field data collection, analyzed results and data interpretation, and contributed to the draft of the manuscript. J. M., C. M., P. I., A. K., M. W., A. A., Z. B., K. T., E. K., P. A.-A., T. T., and F. N. were involved in data collection and participated in laboratory analysis. C. A. N., J. M., D. Y., and M. R. critically reviewed the manuscript. All authors read and approved the final manuscript.

***Acknowledgments.*** We express our gratitude to the populations who participated in this study.

***Financial support.*** The present study was supported by funds from the World Health Organization’s Special Programme for Research and Training in Tropical Diseases (2016/602099-0), which approved the final study design and protocols. C. A. N. was supported by a Wellcome Trust Senior Fellowship in Public Health and Tropical Medicine (202687/Z/16/Z).

***Supplement sponsorship***. The supplement is sponsored by TDR, the Special Programme for Research and Training in Tropical Diseases, based at the World Health Organization.

***Potential conflicts of interest.*** All authors: No reported conflicts of interest.

Presented in part: Sixth Pan African Mosquito Control Association, September 2019, Yaoundé, Cameroon.
