# Supplementary material for: Entomological and Anthropological Factors Contributing to Persistent Malaria Transmission in Kenya, Ethiopia, and Cameroon
Source: J Infect Dis. 2021 Apr 27;223(Suppl 2):S155–70. doi: 10.1093/infdis/jiaa774 (PMC8079137; doi:10.1093/infdis/jiaa774)
Supplement: jiaa774_suppl_Supplementary-Table-S1 [file jiaa774_suppl_supplementary-table-s1.docx]

| Table S1: Characteristics of the study sites | | | | | | |
| --- | --- | --- | --- | --- | --- | --- |
| **Country** | **Locality** | **Coordinates** | **LLINs/IRS** | **Major vectors** | **Secondary/other vectors** | **References** |
| Cameroon | Olama | 3°24’N; 11°18’E | LLINs | *An. moucheti* | *An. paludis; An. ziemani; An. marshallii* | *[*[*15*](#_ENREF_15)*,* [*16*](#_ENREF_16)*,* [*47*](#_ENREF_47)*]* |
|  | Nyabessan | 2°80’N; 10°25’E | LLINs | *An. moucheti; An. gambiae; An. nili* | *An. paludis; An. ziemani; An. marshallii* | *[*[*15*](#_ENREF_15)*,* [*16*](#_ENREF_16)*,* [*47*](#_ENREF_47)*]* |
| Kenya | Chodari | 3^o^7676’N; 39^o^7710’E | LLINs | *An. gambiae , An. arabiensis; An. funestus* | *An. merus; An. squamosus* | [[52](#_ENREF_52), [66](#_ENREF_66)] |
|  | Ngombeni | 3^o^7402’N; 39^o^7721’E | LLINs | *An. gambiae , An. arabiensis; An. funestus* | *An. merus; An. squamosus* | [[52](#_ENREF_52), [66](#_ENREF_66)] |
|  | Mapawa | 3^o^7402’N; 39^o^7721’E | LLINs | *An. gambiae , An. arabiensis; An. funestus* | *An. merus; An. squamosus* | [[52](#_ENREF_52), [66](#_ENREF_66)] |
|  | Ziani, | 3^o^7484’N; 39^o^7207’E | LLINs | *An. gambiae , An. arabiensis; An. funestus* | *An. merus; An. squamosus* | [[52](#_ENREF_52), [66](#_ENREF_66)] |
|  | Makata | 3^o^7323’N; 39^o^7965’E | LLINs | *An. gambiae , An. arabiensis; An. funestus* | *An. merus; An. squamosus* | [[52](#_ENREF_52), [66](#_ENREF_66)] |
|  | Pingilikani | 3^o^7596’N; 39^o^7895’E | LLINs | *An. gambiae , An. arabiensis; An. funestus* | *An. merus; An. squamosus* | [[52](#_ENREF_52), [66](#_ENREF_66)] |
| Ethiopia | Bore Tika | 9°02’00’’N 38°44’48’’E | IRS/LLINs | *An. arabiensis;* | *An. pharoensis ; An. coustani* |  |
|  | Chewaka | 8°26’02’’E 36°19’48’’N | IRS/LLINs | *An. arabiensis;* | *An. pharoensis An. coustani* | [[54](#_ENREF_54), [55](#_ENREF_55), [67](#_ENREF_67), [68](#_ENREF_68)] |
| Abbreviations. IRS : Indoor Residual Spraying; LLINs: Long Lasting Impregnated Nets; | | | | | | |
