# Supplementary material for: Entomological and Anthropological Factors Contributing to Persistent Malaria Transmission in Kenya, Ethiopia, and Cameroon
Source: J Infect Dis. 2021 Apr 27;223(Suppl 2):S155–70. doi: 10.1093/infdis/jiaa774 (PMC8079137; doi:10.1093/infdis/jiaa774)
Supplement: jiaa774_suppl_Supplementary-Data_Caption [file jiaa774_suppl_supplementary-data_caption.docx]

***Supplementary data:*** Table S1: Characteristics of the study sites; Table S2: Species Composition and behavior of anopheline mosquitoes by method of collection in Cameroon, Ethiopia and Kenya; Table S3: indoor hourly malaria exposure of different population categories in Kenya, Ethiopia and Cameroon; Table S4: Circumsporozoites rate of malaria vector collected by other tools than HLC from Kenya, Cameroon and Ethiopia
